# A Major Determinant of Cyclophilin Dependence and Cyclosporine Susceptibility of Hepatitis C Virus Identified by a Genetic Approach

**DOI:** 10.1371/journal.ppat.1001118

**Published:** 2010-09-23

**Authors:** Feng Yang, Jason M. Robotham, Henry Grise, Stephen Frausto, Vanesa Madan, Margarita Zayas, Ralf Bartenschlager, Margaret Robinson, Andrew E. Greenstein, Anita Nag, Timothy M. Logan, Ewa Bienkiewicz, Hengli Tang

**Affiliations:** 1 Department of Biological Science, Florida State University, Tallahassee, Florida, United States of America; 2 Department of Infectious Diseases, Molecular Virology, University of Heidelberg, Heidelberg, Germany; 3 Gilead Sciences, Foster City, California, United States of America; 4 Department of Chemistry, Florida State University, Tallahassee, Florida, United States of America; 5 Department of Biomedical Sciences, Florida State University, Tallahassee, Florida, United States of America; University of Southern California, United States of America

## Abstract

Since the advent of genome-wide small interfering RNA screening, large numbers of cellular cofactors important for viral infection have been discovered at a rapid pace, but the viral targets and the mechanism of action for many of these cofactors remain undefined. One such cofactor is cyclophilin A (CyPA), upon which hepatitis C virus (HCV) replication critically depends. Here we report a new genetic selection scheme that identified a major viral determinant of HCV's dependence on CyPA and susceptibility to cyclosporine A. We selected mutant viruses that were able to infect CyPA-knockdown cells which were refractory to infection by wild-type HCV produced in cell culture. Five independent selections revealed related mutations in a single dipeptide motif (D316 and Y317) located in a proline-rich region of NS5A domain II, which has been implicated in CyPA binding. Engineering the mutations into wild-type HCV fully recapitulated the CyPA-independent and CsA-resistant phenotype and four putative proline substrates of CyPA were mapped to the vicinity of the DY motif. Circular dichroism analysis of wild-type and mutant NS5A peptides indicated that the D316E/Y317N mutations (DEYN) induced a conformational change at a major CyPA-binding site. Furthermore, nuclear magnetic resonance experiments suggested that NS5A with DEYN mutations adopts a more extended, functional conformation in the putative CyPA substrate site in domain II. Finally, the importance of this major CsA-sensitivity determinant was confirmed in additional genotypes (GT) other than GT 2a. This study describes a new genetic approach to identifying viral targets of cellular cofactors and identifies a major regulator of HCV's susceptibility to CsA and its derivatives that are currently in clinical trials.

## Author Summary

Identification of cellular cofactors and their mechanisms of action is a fundamental aspect of virus-host interaction research. Screening of genome-wide small interfering RNA libraries has become an efficient way of systematically discovering cellular cofactors essential for various aspects of viral life cycle. We and others have recently demonstrated that cyclophilin A (CyPA) is an essential cofactor for hepatitis C virus (HCV) infection and serves as the direct target of a new class of clinical anti-HCV compounds, cyclosporine A (CsA) and its derivatives, that are devoid of immunosuppressive function. Here we report the identification of a key regulator of HCV's dependence on CyPA and susceptibility to CsA using a novel genetic screening approach that can potentially be applied to additional cellular cofactors and other viruses. The effectiveness of this approach, termed cofactor-independent mutant (CoFIM) screening, was further supported by results obtained with a parallel CsA-based selection using additional genotypes of HCV. This paper reports a new technology with which we discover and characterize the major determinant of HCV's sensitivity to CyPA inhibitors, which are currently being tested in clinical trials.

## Introduction

Successful completion of the life cycle of a virus depends not only on the function of proteins encoded by the virus but also on cellular cofactors. The availability of genome-scale small interfering RNA (siRNA) libraries and high-throughput screening technology has permitted systematic efforts to discover cellular proteins important for viral infections in cell-culture systems. Typically, proteins with apparent functional relevance to the particular virus are characterized in detail after the discovery, but the mechanisms of action for many other cofactors remain undefined. An important step toward the illustration of the mechanism is to identify the viral agent through which a cofactor functions. Although in rare cases, when small chemical compounds are available whose target is the cellular cofactor, screening for compound-resistant mutant virus can provide valuable information about the viral target, but this approach is not useful for the majority of cofactors. Here we report a new genetics approach, which we designate cofactor-independent mutant (CoFIM) screening, to identify the viral targets of cellular cofactors through the selection of mutant viruses that can replicate in cells where a particular cellular cofactor is knocked down. Because our approach does not rely on prior knowledge of the function of the cofactor or the availability of chemical inhibitors, it may be broadly applicable to cellular cofactors with unknown mechanisms of action.

Various cellular proteins have been implicated in the life cycle of hepatitis C virus (HCV), identified mostly by protein-protein interaction and/or siRNA library screening [1]. A critical role for cyclophilins (CyPs) in HCV replication was first suggested by the direct antiviral effect of cyclosporine A (CsA) [2], [3]. We and others then identified CyPA as the main CyP isoform that serves as an essential cofactor for HCV infection [4], [5], [6] and the principal mediator of CsA resistance [4], [5]. In addition, the peptidyl-prolyl isomerase (PPIase) motif of CyPA has been found to be important for HCV replication [5], [6], [7]. Although CyP inhibitors are currently being studied in clinical trials as a novel class of anti-HCV drugs, the viral target of CsA and the substrate of CyPA's PPIase activity are not well characterized. Nonstructural proteins 5B (NS5B), NS5A, and NS2 have all been proposed to be potential targets of CyPA [8], [9], [10], [11], [12]. In addition, resistance-mapping studies using subgenomic replicons have so far generated a fragmented picture of determinants of CsA susceptibility [5], [9], [12]. In this study, we isolated a mutant JFH-1 full-length virus that escaped the inhibition by shRNAs targeting CyPA. Characterization of the mutant virus revealed a critical dipeptide motif and several surrounding prolines in domain II of NS5A to be the principal modulators of CyP dependence and CsA sensitivity, in a cell culture model of HCV infection (HCVcc) [13], [14], [15], [16].

## Results

### In vitro selection of a HCV mutant strain that is less dependent on CyPA and less susceptible to CsA

**Figure 1:**
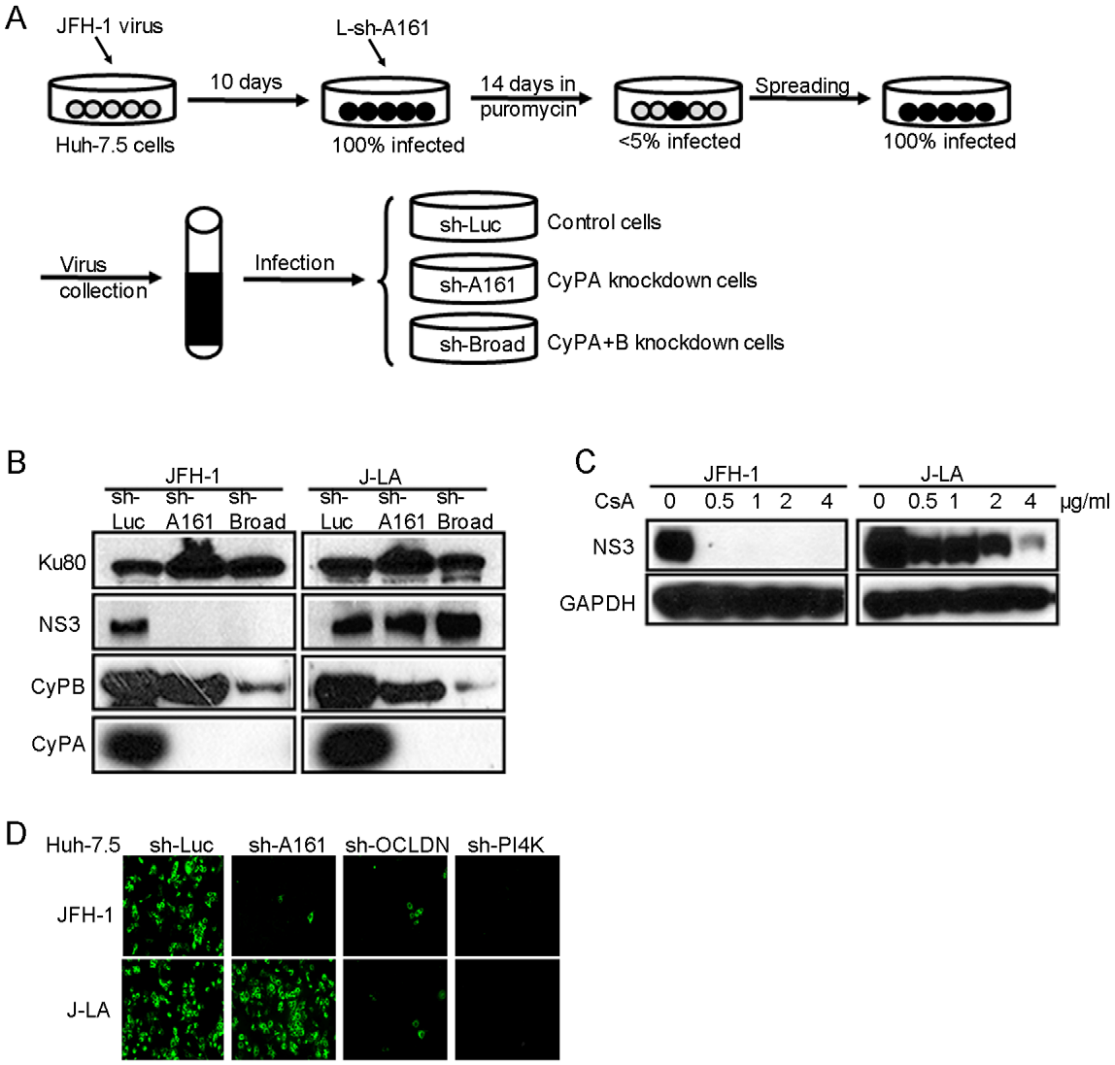
CoFIM selection of a full-length HCV that is less dependent on CyPA. (*A*) A diagram of CoFIM selection #1. Sh-A161 was introduced into infected cells and selected with puromycin. Stable cells were then passaged until all cells became infected. (*B*) The selected mutant virus (J-LA) efficiently infects CyPA and CyPB knockdown cells. Supernatant collected from (*A*) was used to infect cells harboring indicated shRNAs; infected cell lysates were collected 4 days after infection and analyzed by SDS-PAGE and western blotting. A cellular protein, Ku80, was detected as a loading control. (*C*) J-LA is more resistant to CsA treatment than WT JFH-1. Huh-7.5 sh-Luc cells were infected with JFH-1 or J-LA for 24 h before CsA was added to the culture medium. The cells were incubated for 4 more days before being lysed and analyzed for NS3 expression. (*D*) J-LA virus efficiently infects CyPA knockdown cells but not PI4K or Occludin knockdown cells. Equal numbers of Huh-7.5 cells harboring either control or the indicated shRNAs were seeded and infected with either JFH-1 or J-LA virus. Five days after infection, cells were fixed and subjected to anti-NS3 antibody staining. Data shown in (*B*), (*C*), and (*D*) represent two independent experiments.

Although CsA-resistant replicons have been isolated [5], [9], [12], attempts to obtain CsA-resistant full-length HCVcc had been unsuccessful. Because we previously observed a potent block of HCVcc infection of Huh-7.5 cells expressing a small-hairpin RNA (sh-A161) that downregulated CyPA expression by more than 90% [4], we devised a CoFIM selection for CyPA using this shRNA to obtain a HCV mutant that was less dependent on CyPA. In the first selection, we infected Huh-7.5 cells with JFH-1 at a low multiplicity of infection (MOI<0.01) and cultured the cells until 100% of the cells became positive for HCV staining. We reasoned that the relatively long period (10–14 days) of infection spreading time might maximize the diversity of the HCV quasispecies and increase the number of preexisting mutations. We then transduced into the infected Huh-7.5 cells a lentiviral vector expressing sh-A161 (Figure 1A). After a 2-wk antibiotic selection of shRNA-expressing cells, more than 95% of the cells cleared viral infection as a result of suppression of CyPA, but a small percentage (<5%) of cells remained positive for HCV core staining. Continued culturing of these cells for one more week resulted in a population of cells that were 100% infected despite efficient CyPA knockdown, indicating the emergence of a mutant virus that could replicate efficiently with significantly lower levels of CyPA. Viral particles produced from these selected cells were collected and then used to infect Huh-7.5 cells expressing shRNAs directed at firefly luciferase (sh-Luc), CyPA (sh-A161), or CyPA and CyPB (sh-Broad). Although wild-type (WT) JFH-1 could only infect the sh-Luc cell line, the selected virus, designated J-LA, infected all three cell lines with high efficiency (Figure 1B). These results indicated that J-LA was less dependent on CyPA and did not develop the ability to use CyPB as a substitute cyclophilin in the presence of CyPA knockdown. We then measured the CsA sensitivity of JFH-1 and J-LA and observed a greater than 16-fold shift of CsA sensitivity (Figure 1C), consistent with our previous finding that the CsA resistance was correlated with reduced dependence on CyPA in the setting of a subgenomic replicon [4]. The reduced dependence on CyPA was specific, as J-LA virus was not able to infect Huh-7.5 cells containing a shRNA targeting either one of two other essential HCV cofactors (Phosphatidylinositol 4-kinase alpha and Occludin-1) (Figure 1D) [17], [18], [19], [20], [21], [22], [23], [24], [25].

### Mutations in a dipeptide motif in domain II of NS5A are responsible for the reduced dependence on CyPA and the reduced susceptibility to CsA

**Figure 2:**
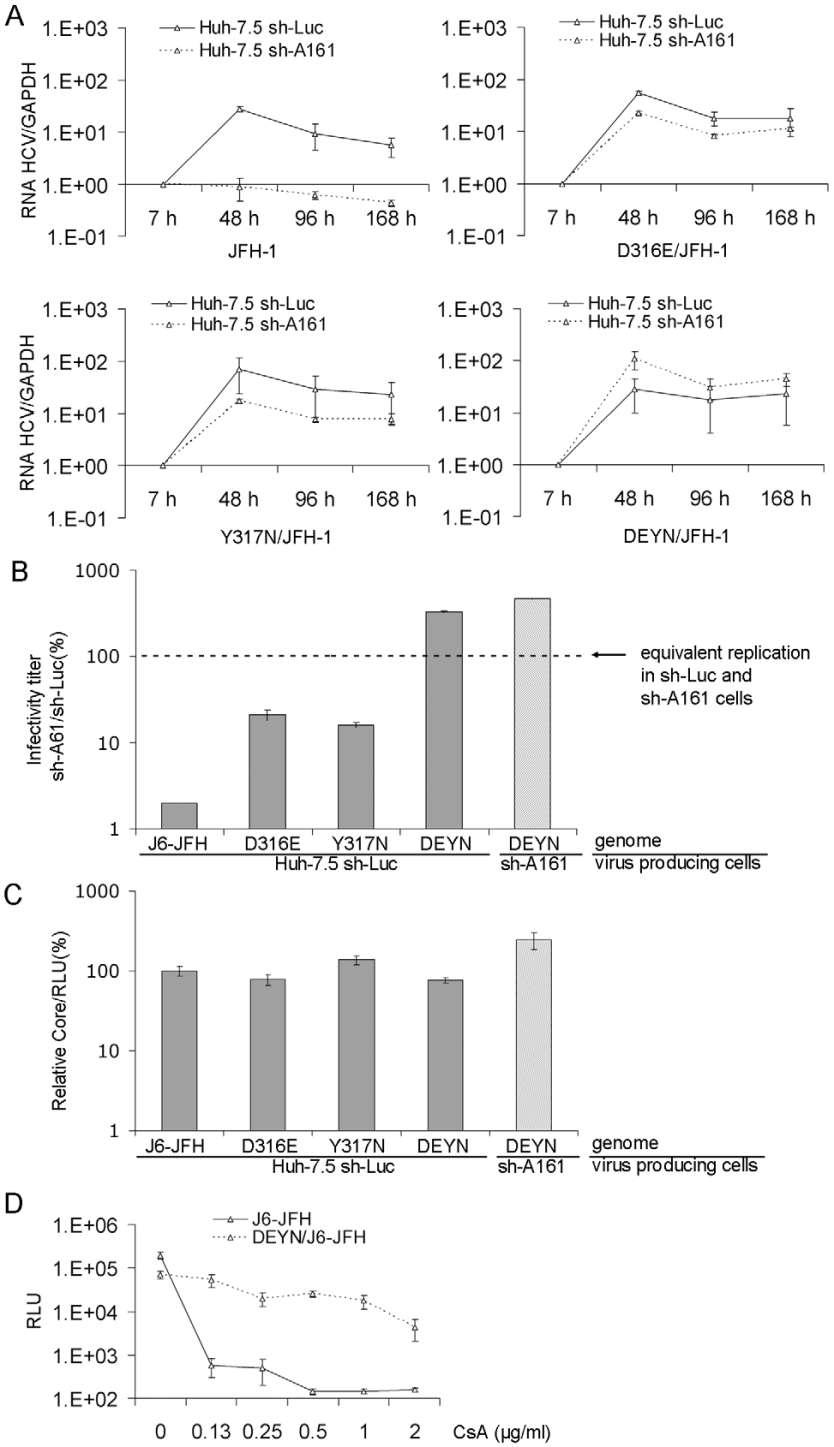
Mutations in a dipeptide motif in NS5A domain II confer CsA resistance and reduced CyPA-dependence. (*A*) Efficient replication of mutant HCV genomes in CyPA knockdown cells. JFH-1 RNA harboring D316E, Y317N, or both was introduced into Huh-7.5 sh-Luc and sh-A161 cells. Total RNAs were extracted 7, 48, 96, and 168 h after electroporation and subjected to quantitative RT-PCR for measurement of HCV and GAPDH RNA. All the data were normalized to the 7 h input. (*B*) Infection of sh-A161 cells with mutant viruses. Infectious viral particles were collected from transfected cells 48–72 hours after transfection and used to infect both sh-Luc and sh-A161 cells. The efficiency of infecting sh-A161 cells was measured as the ratio of infectivity in sh-A161 cells to that in sh-Luc cells for each virus. For the virus harboring D316E/Y317N (DEYN) mutations, particles produced from both sh-Luc (white bars) and sh-A161 (hatched bars) were tested. (*C*) The DE and YN mutations do not significantly impact viral production. Huh-7.5 sh-Luc or sh-A161 cells were electroporated with indicated RNAs. At 72 hours post electroporation, cells were lysed for intracellular luciferase activity and culture media were collected for core ELISA. (*D*) DEYN mutations confer CsA resistance on the J6-JFH genome. Huh-7.5 sh-Luc cells were infected with WT J6-JFH (p7-Rluc2A) or DEYN/J6-JFH (p7-Rluc2A) for 24 h before CsA was added to the culture media. The cells were incubated for 4 more days before being lysed and analyzed for luciferase expression. Error bars in (*A*), (*B*), (*C*), and (D) represent standard derivations of at least two independent experiments.

Three additional independent CoFIM selections (#2, 3, 4 in Table 1) with different parameters produced additional preparations of mutant viruses that were capable of infecting CyPA knockdown cells. The order of HCV and shRNA introduction into the cells was reversed in two of the experiments to reveal whether the same mutant viruses can emerge with preexisting selection pressure. Interestingly, the process took much longer (6 rather than 3 wk) to produce sh-A161-resistant virus when JFH-1 RNA, transcribed in vitro with T7 phage polymerase from a DNA plasmid as template, was electroporated directly into sh-A161 cells, probably because of the lack of diversity and preexisting mutations in the input RNA. Sequence analysis of these mutant viruses revealed related mutations in a dipeptide motif (D316 and Y317) in the domain II of NS5A in all four mutant viral samples. In addition, an I31T mutation in E2 was identified in three of the four selections, whereas the remaining selection revealed a T33A mutation, also in the E2 protein (Table 1). We engineered mutant viruses containing either the E2 mutation I31T or the NS5A mutations and tested their ability to infect sh-A161 cells. The mutant virus containing E2:I31T was not able to infect sh-A161 cells efficiently, and further passage of this virus in sh-A161 cells resulted in the emergence of the NS5A:Y317N or NS5A:Y317H mutation (#5 in Table 1). These data suggested that the E2:I31T mutation was not able to confer reduced dependence on CyPA, consistent with a recent report that this mutation increased infectivity of HCVcc by reducing lipoprotein association with viral particles, a process not known to involve CyPA [26]. The NS5A mutations, however, did confer reduced CyPA dependence on WT virus when engineered into either the JFH-1 (Figure 2A) or the J6-JFH background (Figure S1). Neither WT virus was able to replicate in sh-A161 cells, but mutant viruses harboring D316E, Y317N, or both mutations replicated efficiently in these cells. While the single mutants replicated 5- to 10-fold less in sh-A161 cells than in sh-Luc cells, a combination of the two generated a genome (DEYN) that replicated several fold more efficiently in sh-A161 cells than in the control cells (Figure 2A). The same pattern was observed when the viral genomes were introduced by means of infection rather than electroporation of RNA: the DEYN virus, produced from either sh-Luc or sh-A161 cells, infected sh-A161 cells several fold more efficiently than it did sh-Luc cells, and the single mutants again had an intermediate phenotype (Figure 2B). Of note, approximately same amount of viruses were produced by WT and the mutant genomes in sh-Luc cells (Figure 2C), indicating that these mutations do not significantly impact viral assembly. Furthermore, sh-A161 cells replicating DEYN mutant RNA showed no defect in producing viral particles (Figure 2C, stripe bar) that were able to infect naïve sh-A161 cells (Figure 2B, stripe bar), suggesting that the DEYN virus is capable of completing the entire viral life cycle efficiently in this CyPA knockdown environment. In addition, other selected mutations (Y317H, Y317R) at the Y317 position also conferred reduced CyPA dependence on the J6-JFH virus (Figure S1). Finally, the double mutant (DEYN) exhibited an approximately 20-fold resistance to CsA treatment but remained sensitive to IFN treatment (Figure 2D, Figure S2). Together, these data identify NS5A as a major viral target of CyPA and demonstrate that the DY motif in NS5A is a major determinant for CyPA dependence and CsA susceptibility.

**Table 1:** Mutations identified in JFH-1 full-length viruses^a^ that are less dependent on CyPA for infection.

CoFIM Sel.	Approach	Time	Mutations	
			E2	NS5A
1	CyPA KD in JFH-1–infected cells	3 wk	I31T	D316E^b^ Y317N, D316E+Y317N
2	CyPA + CyPB KD in JFH-1–infected cells	3 wk	I31T	D316E^b^ Y317N, D316E+Y317N^c^
3	Electroporation of JFH-1 RNA into CyPA KD cells	6 wk	T33A	Y317H, Y317R
4	Infection of CyPA KD cells with JFH-1	3 wk	I31T	Y317N
5	Infection of CyPA KD cells with JFH-1 harboring E2:I31T mutation	3 wk	I31T^d^	Y317N, Y317H

a HCV sequences were obtained from intracellular viral RNA.

b Two types of nucleotide mutations (GAC → GAA; GAC → GAG) were found for the D316E mutation.

c Out of 19 bacterial clones sequenced from this selection, 9 contained D316E, 7 contained Y317N, and 3 contained D316E + Y317N.

d Preexisting E2 mutation.

A near-perfect repeat of the peptide containing the DY motif and the four prolines occurs immediately downstream in the JFH-1 NS5A protein (Figure S3A), but we did not recover any mutations in this second DY motif in any of our screens. Moreover, mutation of this second DY motif (D329EY330N) resulted in a replication-deficient virus (Figure S3B). These data suggest a functional divergence of the two repeated DY motifs.

### Mapping the putative proline substrates of CyPA

**Figure 3:**
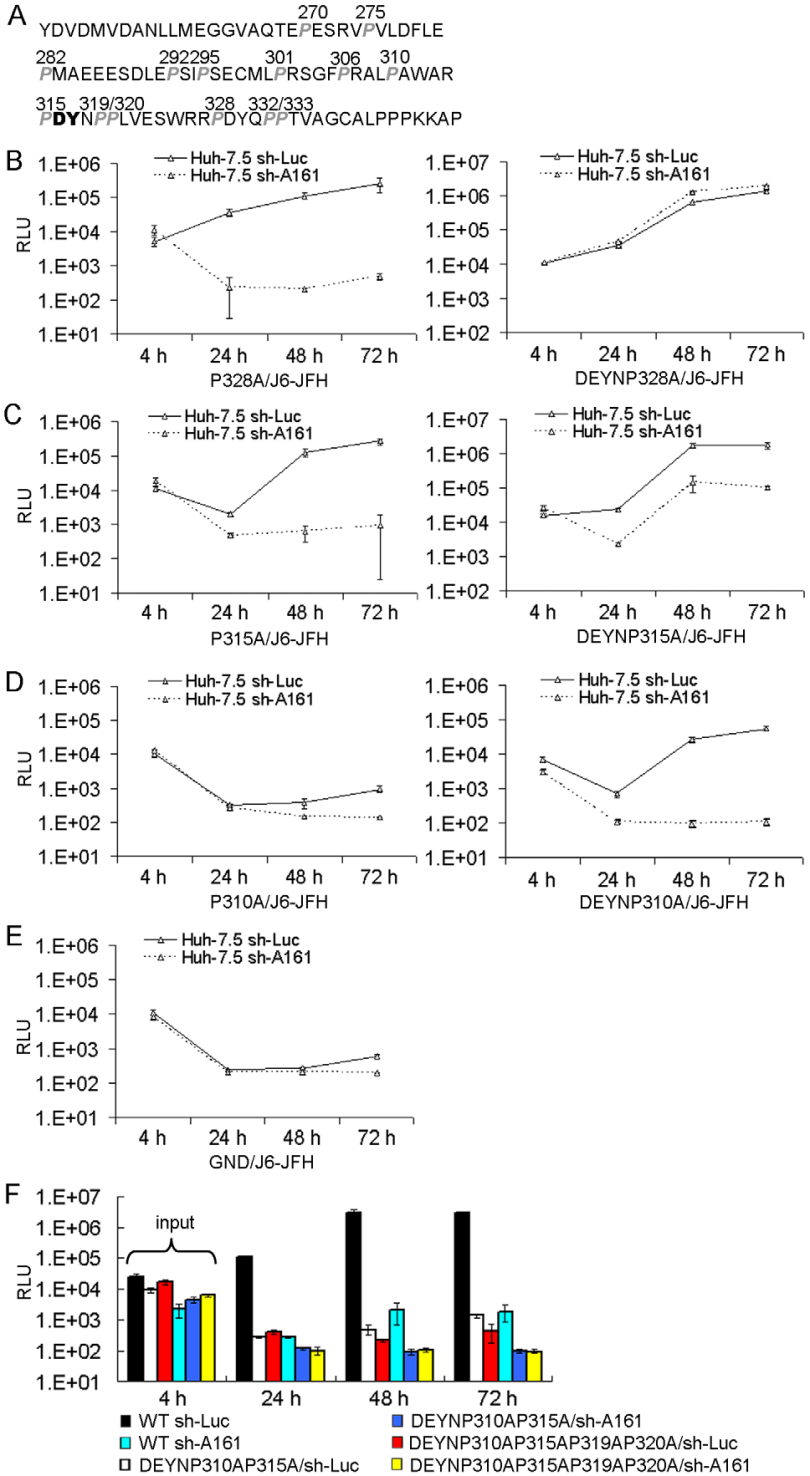
Putative proline substrates of CyPA in NS5A domain II. (*A*) Sequence of NS5A domain II. Each proline was changed to alanine in both WT and the DEYN background, using the J6-JFH genome. Mutant RNAs were electroporated into both Huh-7.5 sh-Luc and sh-A161 cell lines, and luciferase assays were performed at the indicated time points. The prolines were grouped into three categories according to phenotype. Representative replication kinetics of the mutants are shown in (*B*) for group I, (*C*) for group II and (*D*) for group III. The replication profiles of a GND replication defective mutant is shown in (*E*). (*F*) DEYN mutations fail to alleviate the lethal phenotype of double (P310AP315A) or quadruple prolines mutants. Error bars in (*B*), (*C*), (*D*), (*E*), and (*F*) represent standard derivations of two to three independent experiments.

Several independent studies recently demonstrated that the PPIase motif of CyPA is essential for HCV replication [5], [6], [7], suggesting the presence of critical proline residues that serve as substrates for CyPA. We reasoned that the DY motif, which locates to a major CyPA-binding peptide in this domain [11] but not proline themselves, probably confer CsA resistance through the proline substrates of CyPA, and that consequently the mutation of the relevant prolines would affect DEYN's ability to replicate in CyPA knockdown cells. To identify such proline residues, we changed the individual prolines (14 total, Figure 3A) in the domain II of NS5A into alanines, both in the WT and the DEYN background of the J6-JFH genome (Table 2). The mutant genomes were then electroporated into both sh-Luc and sh-A161 cells for measurement of replication capacity. The replication phenotypes of these mutants fell into three categories. Mutations of the first group of prolines had no effect on either the WT's or the DEYN's ability to replicate in both cell lines; i.e., a proline mutant in the WT background would replicate in sh-Luc cells but not in sh-A161 cells, whereas the same proline mutant in the DEYN background would replicate in both cells with high efficiency (Figure 3B). Mutations of the second group of prolines had minimal effect on the WT virus but reversed the replication capacity of the DEYN virus in sh-Luc and sh-A161 cells (Figure 3C); i.e., the DEYN + proline mutant replicated less efficiently in sh-A161 cells than in the control cells. Mutation in the third group, which included only one proline (P310), had the most profound effect: P310A reduced the replication capacity of the WT virus to the level of the GND pol- virus in both cells (Figure 3D left panel, 3E), it also completely abolished DEYN's ability to replicate in sh-A161 cells (Figure 3D, right panel); moreover, it reduced the replication capacity of DEYN virus in the control cells more than any other single proline mutant (Figure 3D, right panel). These data suggested a functional interaction between the DY motif and the four prolines in groups II (prolines 315, 319, 320) and III (proline 310) in determining CyP dependence and CsA susceptibility, and a possible use by CyPA of multiple prolines as substrates on the NS5A protein. The importance of prolines 310 and 315 in HCV replication was further supported by results from double and quadruple mutants in the DEYN background. DEYN was not able to rescue the replication of either the quadruple mutant (with all 4 prolines mutated to alanine) or a double mutant containing P315A and P310A (Figure 3F).

**Table 2:** Alanine-scanning mutagenesis of proline residues in NS5A domain II.

Proline mutation	Replication capacity^a^				
	WT		D316E/Y317N mutations		
	sh-Luc	sh-A161	sh-Luc	sh-A161	sh-A161/sh-Luc^b^
None	+++	–	+++	++++	1.71±0.19
P270A	n.d.	n.d.	++	+++	1.45±0.01
P275A	++	–	++	+++	1.83±0.07
P282A	++	–	+	+	1.51±0.5
P292A	++	–	+++	++++	1.34±0.1
P295A	++	–	++	++++	2.13±0.03
P301A	++++	–	++	++++	2.14±0.01
P306A	+++	–	++	+++	1.19±0.22
P310A	–	–	+	–	***0.01±0.00***
P315A	+	–	++++	–	***0.04±0.01***
P319A	+++	–	++++	+	***0.04±0.01***
P320A	++++	–	++++	+++	***0.57±0.06***
P328A	+	–	+	++	1.45±0.2
P332A	+	–	++	+++	1.32±0.49
P333A	n.d.	n.d.	+	++	1.42±0.02

a Replication capacity of the WT (fold increase from 4 h to day 3) was set at 100%, and the following scales were used: –, 0–10%; +, 11–50%; ++, 51–75%; +++, 76–100%; ++++, >100%.

b The ratio of replication capacity in sh-A161 cells to that in sh-Luc cells was calculated and presented for all the D316E/Y317N mutation–based genomes. Standard errors of two independent experiments are shown.

### DEYN mutations do not affect CyPA-binding or NS5A-NS5B cleavage

**Figure 4:**
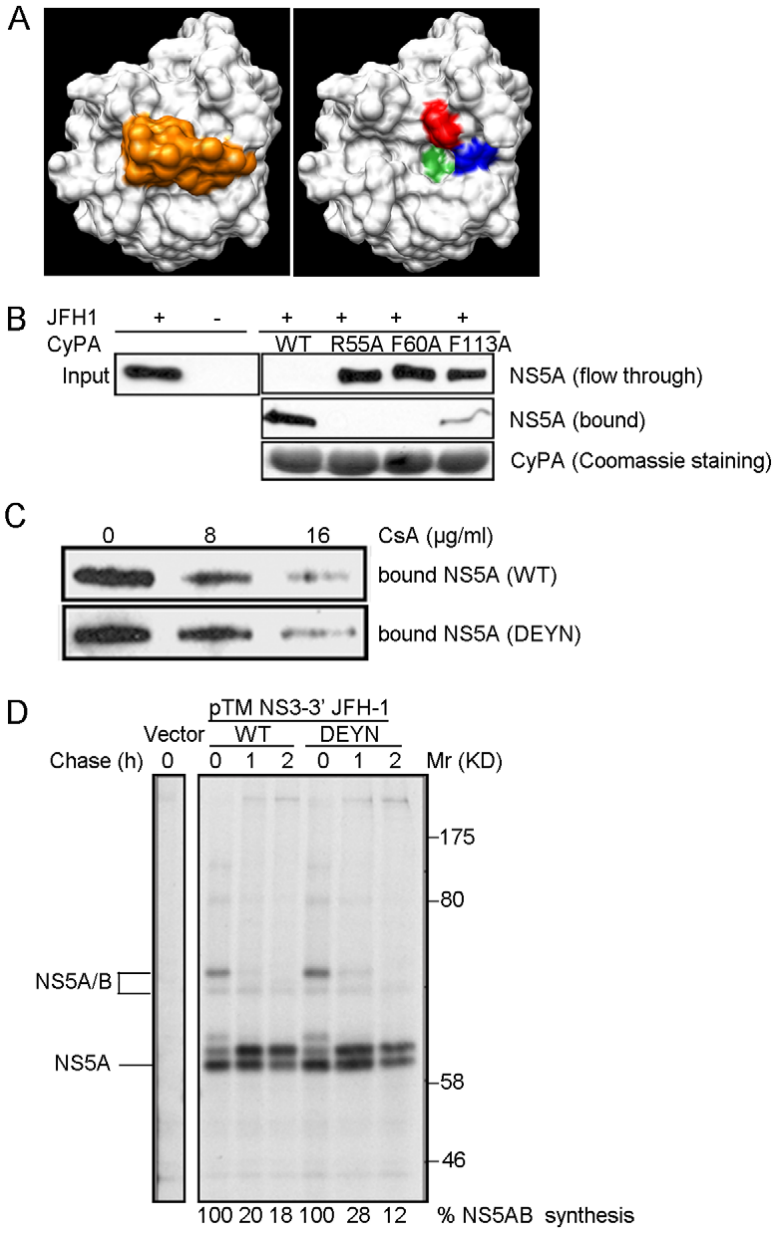
DEYN mutations do not affect NS5A binding or NS5A-5B cleavage. (*A*) Structure of CyPA. Left panel: Space-fill model of CyPA-CsA complex (2RMA) [52]. White: CyPA; orange: CsA. Right panel: residues in the active site of CyPA that are mutated in this study. Red: R55; blue: F60; green: F113. (*B*) In vitro binding of CyPA to full-length NS5A. His-tagged CyPA proteins was incubated with infected cell lysate and then subjected to Ni-NTA column pull-down. CyPA-NS5A complexes were eluted with Imidazole (bound) and then subjected to immunobloting with anti-NS5A antibodies. Result shown is representative of two independent experiments. (*C*) WT and DEYN mutant NS5A protein exhibit similar CsA sensitivity in NS5A binding. Binding reactions were performed as described in (*B*) with increasing amount of CsA present. (*D*) Cleavage kinetics at NS5A-5B site, depicted by pulse-chase labeling and immunoprecipitation. NS5AB protein bands were quantified by densitometry, and we calculated viral protein synthesis by dividing the values obtained for the chase samples by the corresponding values obtained from the 0 h chase samples. Molecular weights are shown at the right; lines point to the respective HCV proteins.

To determine whether the DEYN mutations affect the CsA sensitivity of NS5A's binding to CyPA, we first established a biochemical binding assay for the CyPA-NS5A interaction. Recombinant forms of WT and three mutant CyPA proteins that each contained a single mutation in the active site of the PPIase (Figure 4A) [27] were purified as hexahistidine-tagged proteins and mixed with lysates of JFH-1 infected cells for an affinity pull-down experiment. WT CyPA efficiently bound to full-length NS5A in this assay. The F113A mutant retained approximately 10–20% of NS5A-binding, while mutations R55A and F60A completely abolished NS5A-binding by CyPA (Figure 4B). These results demonstrated the importance of CyPA's PPIase motif in NS5A-binding. We next added increasing amounts of CsA to the binding reaction and observed that the CyPA-NS5A interaction is sensitive to CsA. However, DEYN mutations did not significantly alter the CsA-sensitivity of this interaction (Figure 4C). These data argue against the possibility that the DEYN mutations conferred CsA resistance to the virus by altering CyPA-NS5A binding. We also analyzed the possible connection between CyPA-independence and the phosphorylation status of NS5A. In the biochemical binding assay, the CyPA interacted with both forms (hyper- and basal-phosphorylated) of NS5A to approximately the same extent (Figure S4A). In addition, neither DEYN mutations nor CsA treatment changed the relative ratio of these two phosphorylated forms (Figure S4B). We conclude from these experiments that CyPA's action is probably unrelated to the phosphorylation status of NS5A. We next determined whether the CsA resistance conferred by these mutations was related to altered cleavage kinetics at the NS5A-NS5B junction, as suggested by a previous study [5]. The DEYN mutations in JFH-1 were transferred into a polyprotein expression plasmid, which was then used to express HCV proteins for the measurement of cleavage kinetics. These mutations produced no significant effect on the rate of NS5A-NS5B cleavage (Figure 4D), suggesting that the DEYN mutant identified here uses a mechanism distinct from those of the previously reported cleavage mutants (NS5A:V464A; NS5A:V464L) that could also replicate, albeit to a lower level, in a CyPA-knockdown cell line [5].

### DEYN mutations confer a conformational change at a major CyPA substrate site on the NS5A protein

**Figure 5:**
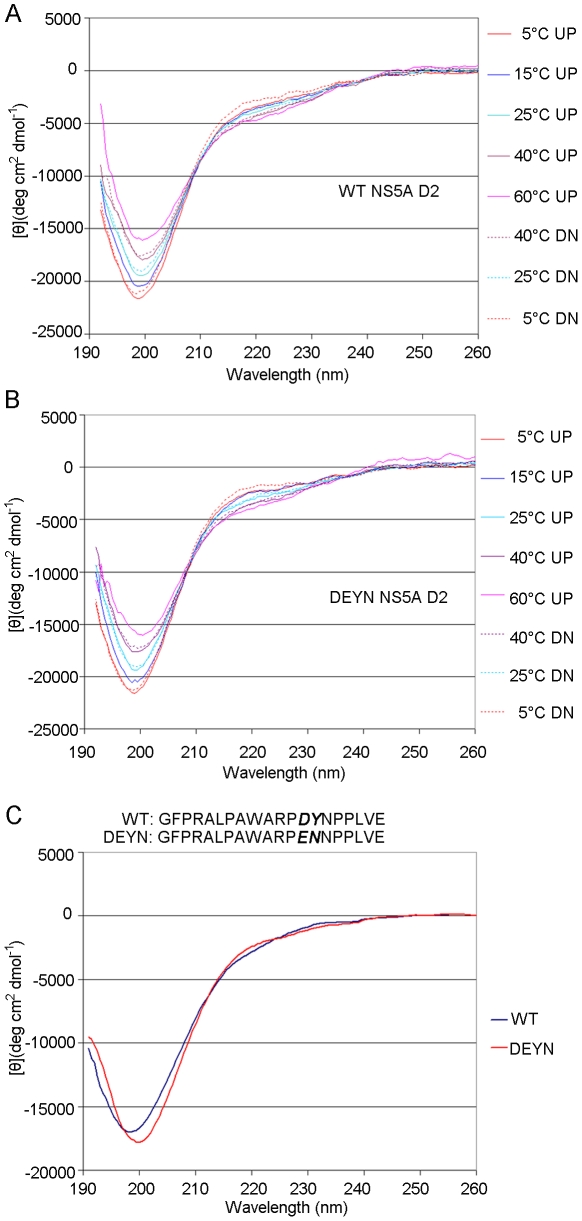
Circular Dichroism analysis of WT and DEYN NS5A D2 and peptides. (*A*) Thermal unfolding of wt NS5A D2 reveals an isodichroic point at approximately 208 nm indicative of a β-turn/extended structure conformational transition [53], [54]. Solid lines represent spectra collected with increasing temperature (UP), with dashed lines showing reversibility of the unfolding process upon subsequent cooling of the wt NS5A D2 sample (DN). (*B*) Similar results were obtained for DEYN NS5A D2. (*C*) The circular dichroism spectra of the WT and DEYN mutant peptides exhibit features characteristic of largely unfolded conformations. The difference in spectral magnitude and a slight shift in the position of the spectral minimum, however, indicate a conformational difference between the WT and DEYN peptides. Result shown is representative of five independent experiments.

**Figure 6:**
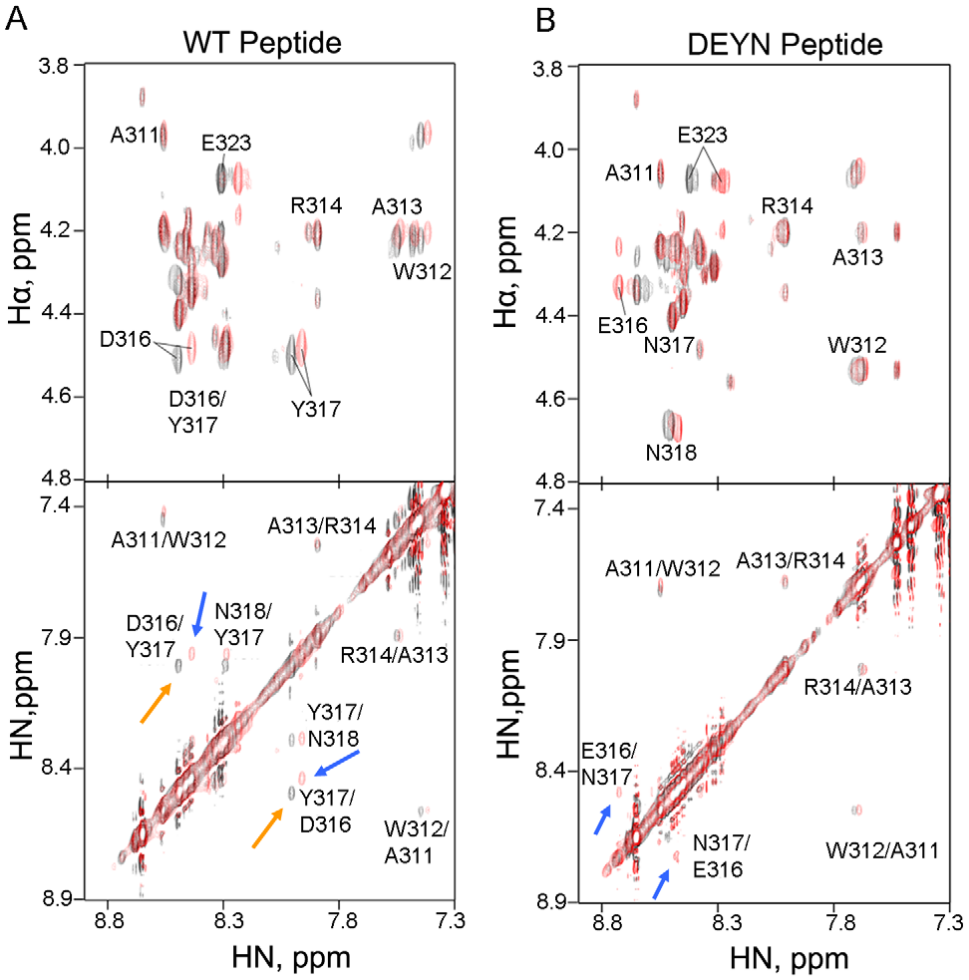
CyPA induces turn conformations in DEYN. Superposition of 400 ms NOESY spectra of (*A*) WT or (*B*) DEYN peptide in the absence (black) and presence (red) of CyPA at a 10∶1 peptide:protein ratio. HN-Hα (top) and HN-HN (bottom) correlations are shown for each sample. WT peptide exhibited sequential HN-Hα and HN-HN NOEs, indicating random coil ensemble of conformers. Addition of CyPA resulted in chemical shift changes but did not significantly alter the NOE pattern or intensities. In contrast, DEYN peptide lacked sequential HN-HN NOEs in D316-N318 in the absence of CyPA, indicating that the EN substitutions increased the population of extended conformers in this region relative to the WT peptide. Addition of CyPA also resulted in chemical shift changes in DEYN peptide and introduced a sequential HN-HN NOE between E316-N317 that is not seen in WT even at the lowest possible contour levels.

The domain II of NS5A has been shown to be largely disordered [28]. We first determined whether this domain, which contains the DEYN mutations and the putative proline substrates, had the potential to adopt different structural conformation. Circular dichroism analysis of recombinant NS5A D2 resulted in spectra indicative of a largely unordered structure (Figure 5). When CD melting experiments were performed, however, an isodichroic point between 209 nm –211 nm was observed in both the WT and the mutant D2 (Figure 5A, B), indicating the existence of at least two different structural conformers in this domain for both proteins. The CD spectra of WT and DEYN mutant proteins, however, were very similar in these melting experiments, indicating that there is no gross structural difference between WT and mutant D2 proteins at the resolution of CD analysis. We then analyzed a 20-mer peptide that contained the DY motif and the four relevant prolines (G304-E323) (Figure 5C) to detect any difference in local conformation introduced by the mutations at this putative CyPA substrate site. Indeed, the spectral amplitude corresponding to the DEYN mutant was more negative and shifted to longer wavelengths compared to the WT peptide (Figure 5C), indicating that the DEYN peptide may adopt a more extended structure with less turn characteristics relative to the WT peptide. To pinpoint the amino acid residues contributing to this structural difference, we characterized each peptide using two dimensional NMR spectroscopy. Specifically, we used 2D NOESY spectra, in which cross peaks arise between protons that are close in space (generally <5 Å). It is well known that helical or turn and β-strand or extended conformations in peptides yield distinct NOE patterns based on ^1^H-^1^H distances in these secondary structures. While the WT peptide adopts a mostly random coil structure, NOE data clearly indicated a more extended structure between residues 316 and 318 for the DEYN peptide. Specifically, we observed sequential αN and NN NOEs for each non-proline residue in the WT peptide, indicating that this peptide adopts a conformational ensemble containing both extended and turn-like conformations (Figure 6A). Interestingly, the amide resonances of residues A311 and W312 exhibited large up-field shifts for a small, random-coil peptide, consistent with the unusual resonance shifts of the A311 and W312 residues observed in a full-length D2 construct [11], suggesting that the peptide may adopt similar conformations as the NS5A protein. We observed sequential αN NOEs for each non-proline residue in DEYN and sequential NN NOEs between residues A311-R314 but did not see sequential NN NOEs between E316-N318 (Figure 6B). This suggests that the DE and YN substitutions bias the peptides towards more extended, β-strand-like conformations compared to the WT sequence.

We also determined if CyPA acted on these peptide substrates differently because of the structural difference that we observed. Adding either the WT or the DEYN peptide to ^15^N-labeled CyPA significantly perturbed the chemical shifts for the residues in the same regions of CyPA (Figure S5) indicating that these two peptides bound to the same region of CyPA, consistent with equivalent binding in the biochemical binding assay. Adding a catalytic amount of CyPA (∼1∶10) to the peptides caused chemical shifts in the following residues: W312, A313, D/E316,Y/N317, E323 (Figure 6, top). With the exception of E323, the peptide residues that shifted upon addition of CyPA were all located between P310 and P320, further supporting the notion that the segment containing these four prolines is a major CyPA substrate relevant for HCV replication. Moreover, we observed a sequential NN NOE between D316-Y317 in WT peptide with or without CyPA, indicating the presence of a turn formation between these residues that is not dependent upon CyPA (Figure 6A, bottom). In contrast, NOE analysis of DEYN peptide showed the absence of an E316-N317 NN NOE, suggesting a reduced tendency for turn formation in the absence of CyPA. Adding a catalytic amount of CyPA to the DEYN peptide induced the formation of a similar turn between residues E316-N317 as evidenced by the presence of an E316-N317 NN NOE (Figure 6B, bottom). These data further validate the DY motif as a major modulator of CyPA's action.

### The major CsA sensitivity determinant is conserved in other genotypes and isolates

**Figure 7:**
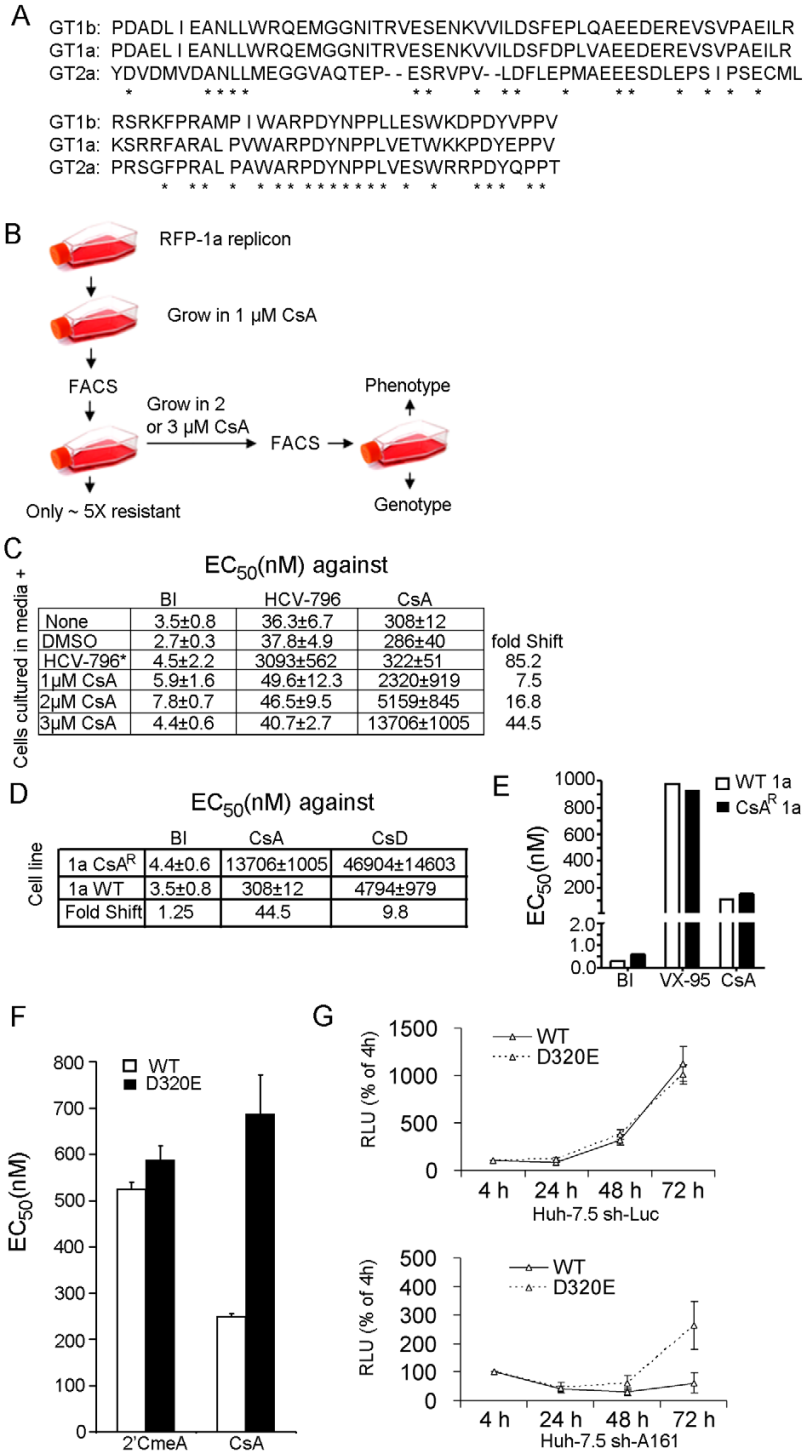
Critical role of NS5A aspartate 316/320 in CsA activity extends to GT 1a. (*A*) Alignment of NS5A domain II sequences from Con1 (GT 1b), H77 (GT 1a), and JFH-1 (GT 2a). (*B*) A diagram of selection of CsA-resistant HCV genotype 1a replicon. Genotype 1a replicon cells were selected by serial passaging in the presence of a selectable marker and either the specified antiviral or a comparable volume of solvent. (*C*) Specific resistance by the selected cells to the selection drug used. The degree to which the EC_50_ of CsA was shifted corresponded to the concentration of CsA in which the cells were selected. The EC_50_ of the nonnucleoside NS5B inhibitor HCV-796 is only shifted for the cells selected in HCV-796. (*D*) Replicon cells selected in 3 µM CsA were cross resistant with a CsA analog that inhibits CyPA but does not bind calcineurin. (*E*) “Supertransfection” of CsA-resistant replicon cells with a WT 1b replicon showed that resistance to CsA is conferred by mutations in the replicon RNA rather than in the host cell. (*F*) The D320E mutation, engineered into a WT 1a replicon in isolation, conferred resistance to CsA but not to 2′CMeA. Error bars represent standard derivations of three independent experiments. (*G*) Transient replication of D320E 1a replicon in sh-A161 cells. A GT1a replicon containing a *Renilla* luciferase reporter gene was used for this assay. The replication capacities of WT and D320E replicons were measured in both Huh-7.5 sh-Luc and Huh-7.5 sh-A161 cells.

The putative peptide substrate of CyPA, centered around the DY motif, is the most conserved region in NS5A domain II of GT 1a, 1b, and 2a (Figure 7A). To determine whether this major determinant of CsA sensitivity that we identified in GT 2a is functionally conserved across genotypes, we conducted a CsA-resistance selection in a GT 1a replicon. Using a GT 1a replicon labeled with red fluorescent protein (RFP), we isolated resistant cells with high replication levels in the presence of CsA, a NS5B inhibitor (200 nM of HCV-796), or an equivalent volume of solvent (DMSO) (Figure 7B). The sensitivity of the selected cells to three antiviral molecules, the protease inhibitor BILN-2061, HCV-796, and CsA, was measured (Figure 7C). All cell lines were equally sensitive to BILN-2061 and those selected in HCV-796 were 74-fold less sensitive to HCV-796 but remained as sensitive to CsA. In contrast, the replicon cells selected in the presence of CsA exhibited specific resistance to CsA, and the levels of resistance directly tracked the concentration of the compound applied during selection.

The CsA-resistant replicon was also cross resistant to CsD, a CsA derivative that is a potent inhibitor of CyPA with dramatically reduced calcineurin binding [29], indicating that the resistant replicon overcame CyPA inhibition rather than calcineurin recruitment (Figure 7D). To determine the source of CsA resistance, we tested the CsA sensitivity of a naïve GT 1b replicon in the CsA-resistant cells. A WT 1b replicon with a *Renilla* luciferase reporter was transfected into the GT 1a replicon cells selected at 3 µM CsA. The sensitivity of the 1b replicon to CsA is determined by monitoring of the luciferase signal. The 1b-luciferase replicon was equally sensitive to CsA in WT or CsA-resistant 1a-RFP replicon cells (Figure 7E). This result demonstrated that the CsA resistance was driven by mutations in the replicon and not in the host cell.

A D320E mutation, which is the equivalent of the D316E mutation in JFH-1, was observed in CsA-resistant cell lines selected with both 2 µM (50% of the bacterial clones sequenced had the mutation) and 3 µM CsA (100% of the bacterial clones sequenced had the mutation) but not in the control cell lines. When the D320E mutation was engineered into a WT GT 1a replicon, it conferred a statistically significant 2.7-fold shift in EC_50_ of CsA but no shift in the EC_50_ of the control antiviral compound 2′CmeA (Figure 7F). In addition, the D320E 1a replicon was able to replicate in sh-A161 cells, albeit to a lower level than in the control cells, while the WT 1a replicon could not (Figure 7G). Replication in control cells was not affected by the DE mutation.

**Figure 8:**
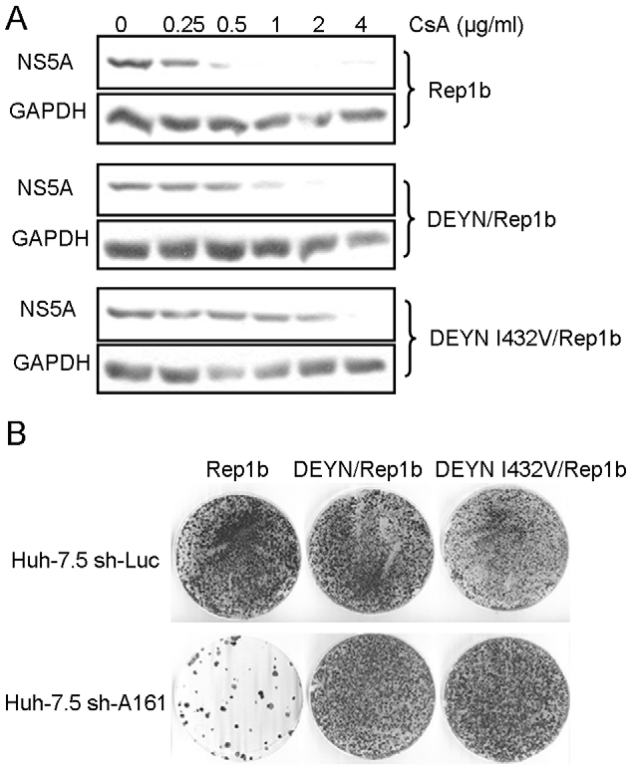
The NS5A DY motif confers CsA resistance and reduced CyPA dependence in a GT1b replicon. (*A*) The D320E/Y321N mutations increased CsA resistance level of a Con1 replicon with or without a NS5B mutation associated with CsA resistance. The replicon cells were treated with CsA for 4 days and then lysed for analysis of NS5A expression with western blotting. Data shown represent two independent experiments. (*B*) DEYN-containing Con1 replicons can efficiently replicate in CyPA knockdown cells. Colony formation assays were performed in both control and knockdown cells with WT and mutant RNA genomes.

We next determined whether the DEYN mutations could also increase CsA resistance in the GT 1b background. We have previously shown that a point mutation in the NS5B gene of a GT 1b replicon (NS5B:I432V) could confer a low level (1.5 fold) of CsA resistance [9]. Combining DEYN with the NS5B mutation generated a mutant replicon (DEYNI432V/Con1) that was significantly more resistant (>4 fold) (Figure 8A). In addition, either DEYN alone or DEYN combined with NS5B:I432V conferred the ability of the Con1 replicon to efficiently replicate in sh-A161 cells in a colony-formation assay (Figure 8B). These data, together with recent reports of the D320E mutation in CsA-resistant GT 1b replicons [30], [31], strongly suggest that the DY motif represents key residues in a functionally conserved determinant of CyPA-dependence and CsA susceptibility across genotypes.

## Discussion

Using a newly developed genetic-selection approach, we obtained for the first time a full-length, CsA-resistant HCV genome that can replicate more efficiently in CyPA-knockdown cells than in control cells. We also identified a specific dipeptide motif that is a major controller of CyPA dependence and CsA susceptibility. Mutation of this DY motif not only renders HCVcc less dependent on CyPA and less sensitive to CsA treatment but also changes the conformation of the NS5A peptide that binds to CyPA. In addition, four prolines that represent putative substrates of CyPA were mapped to a contiguous peptide surrounding the DY motif with the most critical proline located six amino acids upstream of the aspartic acid residue.

Increased RNA binding by HCV replicase and delayed NS5A-NS5B cleavage have been proposed as potential mechanisms of CsA resistance [5], [30], correlated with reduced dependence on CyPA [4]. The mechanism of the DEYN mutations, however, appears to be distinct from either of these, as no difference was observed in either CyPA binding or NS5A-NS5B cleavage. The functional mapping and biophysical characterization of the putative CyPA substrate on NS5A D2 suggests the following molecular mechanism for DEYN-conferred CsA-resistance and reduced CyPA dependence: The WT NS5A D2 structure is largely disordered, containing a mixture of turn-like and more extended conformations at the CyPA substrate site. *Cis* Xaa-Pro peptide bonds further reduce the population of extended conformers in this region. CyPA catalyzes *cis-trans* peptide bond interconversion in NS5A, affecting the population of turn and extended conformers. We propose that the latter are required for the normal biological function of NS5A as part of a replicase component. DEYN mutations cause the structural shift toward this more extended form, reducing the dependence on CyPA to induce such a structure. Of note, aspartate, present in the WT sequence, is a strong N-capping residue for helix formation and mutation to glutamate reduces the helical propensity for these residues, increasing the population of structure conformers with less turn features in the DEYN mutant compared to the WT. It is interesting that CyPA introduces turn formations in the DEYN peptide, presumably through mass action due to the larger population of the extended conformers in this mutant.

The DEYN mutations confer the most marked CyPA-independent phenotype reported thus far and do so in a full-length virus with all viral proteins present. NS2 has recently been shown to increase CsA sensitivity in either the full-length or a replicon background [5], [10]. We did not identify any mutations in the NS2 region in our screens, and the DEYN mutation conferred resistance both in the presence (full-length) or the absence (NS3-NS5B subgenomic replicon, Figure S6) of NS2. In addition, the resistance-conferring mutations that we previously identified in NS5B of a GT 1b replicon [9] were not required for DEYN to confer reduced dependence on CyPA, suggesting that the NS5A DY motif is the primary regulator of CyPA dependence and CsA susceptibility. Note that residual amounts of CyPA exist in Huh-7.5 sh-A161 cells [4], and the DEYN mutant virus is inhibited by CsA at high concentrations (>8 µg/ml), suggesting that either the remaining CyPA or other minor contributors to CsA sensitivity such as other CyP isoforms expressed at low levels [4], [32] facilitate the replication of the DEYN virus in sh-A161 cells. Of note, sh-A161 only inhibits the expression of CyPA, but not other CyP isoforms (Figure S7). To address definitively the issue of CyPA independence, a CyPA-knockout cell line similar to the one generated by Braaten et al. [33] that is also susceptible to HCV infection may be necessary. In this regard, we propose to apply the CoFIM designation to viral mutants with either complete independence or reduced dependence on cellular cofactors.

Proline residues in NS5A that are important for HCV replication have been identified previously, both in GT1b [34] and GT2a [35]. In a cell-culture adapted GT1b replicon, two prolines (P314 and P324) in domain II that are equivalent to P310 and P320 identified in this study were found to be critical for colony formation of the replicon. In addition, several residues in close proximity to these prolines were also essential for replication [34]. It would be interesting to determine whether mutation of these residues can influence CyPA-binding or the related conformation change of NS5A. Finally, a polyproline motif (PP2.1) that locates to the low-complexity sequence [36] between domains II and III of NS5A can regulate both RNA replication and viral assembly [35], raising the possibility of additional CyPA substrates outside NS5A domain II. We speculate that the isomerization of one or more of these prolines of NS5A by CyPA is required for the proper function of NS5A, which is currently not understood, in the life cycle of HCV.

CsA sensitivity appears to vary in different genotypes [37], [38]. An independent selection with a GT 1a replicon identified D320E, which is equivalent to D316E in JFH-1, as an important contributor to CsA resistance. Similar observations have been obtained with GT 1b replicons whereby the proline residues surrounding the D320 residue are also important for replication of the 1b genotype [30], [31], [34]. Analysis of the HCV sequences deposited at the European HCV Database (euHCVdb) revealed that the frequency of changes at both the DY motif and the four prolines in natural isolates are low (Table 3). The most variable position is NS5A 316 and its equivalent position, which had substitutions in only 22 out of 2764 isolates. And although there are some isolates that harbor either the D to E or the Y to N change at the NS5A DY motif, we did not find a single natural isolate that contains both changes. To further address the in vivo relevance of the DY motif, it would be important to determine whether mutations at this site can emerge from patients in clinical trials treated with CsA derivatives such as DEBIO-025, NIM811, or SCY-635 [39], [40], [41].

**Table 3:** Naturally occurring substitutions in HCV NS5A protein sequences.

Position	JFH-1 Residue	Subst. a	No.^b^	Accession number ^C^	Total^d^	Perc.^e^
310	P	A	1	AJ278830 (1a)	10 (A,S,L)	0.10
315	P	A	3	EU155328 (1b)EF407458 (1b)AF207757(1b)	10 (A,S,L,T)	0.30
316	D	E	7	EF407500 (1b)AF033364 (1b)AF033365 (1b)AJ133098AJ133099AF238486 (2b)AF238481 (2a)	22 (G,S,E,N,Y,A)	0.32
317	Y	N	2	DQ491996 (1b)Y12083 (6a)	9 (N,H,C,D,N)	0.22
317	Y	H	1	AF033361 (1b)	9 (N,H,C,D,N)	0.11
319	P	A	0	n.a.	1 (L)	0.00
320	P	A	0	n.a.	1 (V)	0.00

a Substitution described in this paper.

b Total number of sequences with the substitution described in this paper.

c Accession number and genotype of sequences with the substitution described in this paper.

d Total number of sequences with variation at the this position.

e Percentage of naturally variants that are identical to the substitution described in this paper.

So far as we know, this is the first report of an shRNA-based selection of viral mutants with reduced dependence on a cellular cofactor. The general steps of Co-FIM selection starts with the identification of a cellular cofactor, suppression of which would lead to viral inhibition. The next step would be the selection of viral variants that can replicate in the constant pressure of shRNA-mediated knockdown of the cofactor. This can be done either by introducing viral particles or genomes into stable knockdown cells (selection # 3, 4, and 5) or transducing infected cells with lentiviral vectors expressing shRNAs (selection #1 and 2). Sequencing and identification of mutations in the selected viruses are then followed by standard reverse genetics approaches to verify the effect of the mutations. Obviously, a cellular cofactor that is also essential for cell survival would not be amenable for this selection. Also, it might be very difficult for a virus to bypass entire cellular pathways that are important for its replication. For HCV, these may include membrane-reorganizing factors and proteins involved in lipid metabolism, to name a few.

In addition to target identification, the CoFIM screen may also reveal novel cellular pathways involved in the viral life cycle and identify cofactor hierarchy. For example, cross-resistance to knockdown of multiple cofactors may place distinct factors in the same pathway for viral infection and reveal previously unidentified interactions. The strong agreement of our shRNA-based results with those from independent compound-based selection indicates that CoFIM screening may be applicable to a wide range of cellular cofactors in the absence of small-molecule inhibitors or knowledge of mechanism.

## Materials and Methods

### Cell lines, replicons, viruses, antibodies, and compounds

Huh-7.5 cells and J6-JFH (p7-RLuc) were provided by Charles M. Rice. JFH-1 constructs were provided by Takaji Wakita. Huh-7.5 sh-Luc, sh-A161, and Huh-7-Lunet/T7 cells have been described previously [4], [5]. The genotype 1b–PI-luc replicon, used for the supertransfection experiment, and genotype 1a replicons have been described previously [42]. Monoclonal antibodies against JFH-1 NS3 and NS5A were made in house. Compounds BILN-2061 and 2′CmeA were purchased from Acme Biosciences (Palo Alto, CA), HCV-796 from Curragh Chemistries (Cleveland, OH), IFN-α from Sigma-Aldrich (St. Louis, MO), and CsD from Enzo Life Sciences International, (Plymouth Meeting, PA).

### CoFIM selection of sh-A161 resistant JFH-1 virus: transduction route

Huh-7.5 cells were infected with JFH-1 virus at MOI of <0.01 for 4 h. The input virus was removed, and the cells were cultured for 10–14 days, during which a small sample was removed every 3 days for immunofluorescence staining for HCV core and NS3 until all cells were positive for HCV. The infected cells were then transduced with lentiviral vector sh-A161 and were selected in the presence of 1.2 µg/ml puromycin for 3 wk. We first examined the stable cells for CyPA/CyPB with western blotting to confirm knockdown and then examined them for presence of HCV-positive cells by NS3-staining. The cells were then cultured for another week, during which a small sample was removed by every 2 days for determination of the percentage of infected cells. Supernatant containing mutant viruses was collected once all the cells again became 100% infected.

### CoFIM selection of sh-A161 resistant JFH-1 virus: infection/electroporation route

Huh-7.5 cells stably expressing sh-A161 were challenged with JFH-1 viral particles or genome RNA transcribed in vitro. The cells were then cultured for 3–6 wk, during which a small sample was removed every 3 days for NS3 staining. Supernatant containing mutant viruses was collected once >90% of the cells became positive for HCV NS3.

### Selection of CsA-resistant genotype 1a replicon

After 40 days of serial passaging in media supplemented with increasing concentrations of CsA, cells fluorescing in the top 15% of the population were isolated by flow cytometry. After outgrowth in the high concentration of CsA, RNA isolation, RT-PCR, and sequencing were performed by Tacgen (Hayward, CA). To determine drug sensitivity, we seeded stable replicon cells in 96-well plates at a density of 5×10^3^ cells per well or transiently transfected replicon cells at 1.3×10^4^ cells per well and treated them with compounds for 3 days, after which the cells were assayed for luciferase or NS3 activity [43]. Activity levels were converted into percentages relative to the untreated controls (defined as 100%) and data were fit to the logistic dose response equation y (a/(1+(x/b)c) with XLFit4 software (IDBS, Emeryville, CA).

### In vitro binding of CyPA and NS5A

Binding of recombinant His-CyPA to NS5A was performed using total lysate collected from Huh 7.5 L-Luc cells containing either the JFH-1 virus or full-length JFH-1 genomic replicons (pFGR-JFH-1 WT or pFGR-JFH-1 DE/YN). Lysates, generated by lysing 8×10^5^ cells in 600 µl of IP buffer (50 mM Tris-HCl, pH 8.0, 150 mM NaCl, 1 mM EDTA, 0.5% NP-40, 1 mM PMSF, 1 mM DTT, 1x protease inhibitor cocktail), were incubated with His-select Nickel affinity gel (Sigma) prior to binding. Three-hundred micrograms of His-CyPA protein was preincubated with increasing amounts of cyclosporine A for 30 min at 4°C. The recombinant protein was then mixed with 50 µl of cleared lysate and incubated at 4°C or 1 hour. Freshly equilibrated resin was then added to each sample and allowed to bind for 30 min at 4°C. Beads were pelleted at 5000×g for 1 min and the supernatant removed. Three washes were performed using wash buffer (50 mM sodium phosphate, 10 mM imidazole, 250 mM sodium chloride, pH 8.0) followed by elution with wash buffer containing 250 mM imidazole. The flowthrough and eluted (CyPA-bound) fractions were then analyzed by SDS-PAGE and western blotting.

*RT-PCR and sequencing.* Sequences of primer sets used in RT-PCR reactions to amplify JFH-1 genome are available upon request. PCR products were sequenced directly or as cloned inserts in a pCR2.1-TOPO vector (Invitrogen, CA).

### In vitro transcription, electroporation, HCVcc production, and quantitative RT-PCR

These methods have been described previously [4], [42].

### Pulse-chase labeling

The metabolic labeling and immunoprecipitation were performed essentially as previously described [5]. Huh-7 Lunet/T7 cells were transfected with subgenomic NS3 to NS5B JFH-1 expression constructs derived from the WT or DEYN mutant or with the empty vector (pTM1-2). After 22 h, cells were starved for 1 h in methionine/cysteine-free medium and then pulse-labeled for 1 h with [^35^S] methionine/cysteine (150 µCi/ml Express Protein labeling mix). Cells were lysed immediately (0) or incubated with nonradioactive medium for 1 or 2 additional hours (chase). Immunoprecipitation was performed with a monoclonal NS5A-specific antibody. Samples were processed by SDS-PAGE (8%), followed by fluorography and autoradiography.

### Mutagenesis

Site-directed mutagenesis was carried out with a QuikChange kit (Stratagene, CA). Sequences of mutagenesis primers for all the prolines and the DY motif are available upon request. Fragments containing the mutated NS5A sequence were also cloned into a pTM NS3-3′ plasmid to produce subgenomic NS3 to NS5B JFH-1 expression constructs containing the corresponding mutations (pTM NS3-3′DEYN JFH-1).

### Replicon assays

One microgram of in vitro transcribed WT and mutant RNAs were electroporated into 4×10^6^ Huh-7.5 cells stably expressing shRNAs. For the 1a replicons, replication was measured transiently at 4, 48, 72 and 96 hrs post-electroporation. The 1b replicons were allowed to form colonies after three weeks of selection and the resulting colonies were stained with crystal violet. CsA treatment of the stable 1b replicon cell lines were performed as described previously [9].

### Luciferase assays

Luciferase assays was performed according to the manufacturer's instructions for the Dual-Glo Luciferase Assay System from Promega (Madison, WI) with two exceptions: the Luciferase Assay System was used for the Genotype 1b supertransfection experiments, and the *Renilla* Luciferase Assay System was used for the Genotype 1a D320E mutant. Of note, the J6-JFH-1 reporter virus carries a *Renilla* luciferase gene that is not a target of sh-Luc, which is directed against the firefly luciferase gene.

### Core ELISA

HCV core ELISA was performed according to the manufacturer's instructions for the HCV Antigen ELISA kit (Ortho-Clinical Diagnostics, Japan).

### Circular dichroism spectroscopy

Circular dichroism data were collected with a Jasco J-810 spectropolarimeter (Jasco Inc., MD) equipped with Peltier temperature control. The spectra were recorded as averages of 5 scans in 20 mM sodium phosphate, 50 mM NaCl, pH 6.5 buffer at 0.1-nm resolution and 0.05-cm path length. The wt and DEYN NS5A-D2 protein (6.3 µM) and peptide (50 µM) sample concentrations were determined by absorbance at 280 nm and the following extinction coefficients: 14,105 M^−1^/cm^−1^ and 15,595 M^−1^/cm^−1^ for DEYN and wt protein and 5500 M^−1^/cm^−1^ and 6990 M^−1^/cm^−1^ for DEYN and wt peptides, respectively. The NS5A-D2 thermal melt data were collected at a scan rate of 15°C/h with a 10-min equilibration time prior to collection of wavelength spectra at 5, 15, 25, 40 and 60°C. All data were acquired in at least triplicate, with baseline correction and no curve smoothing. The circular dichroism instrument was calibrated with ammonium (+)-camphor-10-sulfonate by a two-point calibration method described previously [44].

### NMR analysis

A synthetic WT peptide corresponding to residues 304-323 of NS5A (304GFPRALPAWARPDYNPPLVE323) and a peptide with the double mutations D316E and Y317N were obtained from NEO BioScience (Cambridge, MA). Purity was determined to be greater than 98% by high-performance liquid chromatography and mass spectrometry. Peptide NMR spectra were collected on 1 mM samples in aqueous buffer at 4°C on a Bruker Avance spectrometer operating at 700 MHz. Total correlation (TOCSY) spectra were collected using a clean-DIPSI mixing sequence (100 ms mixing time) [45] with excitation sculpting solvent suppression [46] as 2048×256 complex matrices with spectral width of 7692.3 Hz in both dimensions. NOESY spectra (400 ms mixing time) were collected under identical spectrometer conditions using excitation sculpting solvent suppression [46]. The indirect dimensions were extended during processing using linear prediction or covariance techniques [47], [48]. The expression and purification of ^15^N-labeled human CyPA was carried out as previously described [11] with the additional purification step of high-performance liquid chromatography (Superdex 75, GE Healthcare). ^15^N-labeled CyPA (∼0.5 mM) was exchanged into NMR buffer (50 mM NaH_2_PO_4_/Na_2_PO_4_, pH 6.3, 40 mM NaCl, 2 mM EDTA, and 1 mM DTT), and peptide was added sequentially to produce the desired final molar equivalents. 2D ^1^H,^15^N-HSQC NMR spectra [49] were acquired at 22°C on a Bruker Avance spectrometer operating at 500 MHz ^1^H frequency. 2D spectra were collected as 1024×200 complex matrices with spectral widths of 833.33 Hz and 2500 Hz in the ^1^H and ^15^N dimensions, respectively, from 16 scans per complex t_1_ point. Spectra were processed with nmrPipe [50] and analyzed with NMRView [51].

## Supporting Information

Figure S1 Mutations at the DY motif confer reduced CyPA dependence in the J6-JFH(p7-Rluc2A) background. Mutant RNAs were transfected into Huh-7.5 sh-Luc and sh-A161 cells by electroporation. Cells were then collected at the indicated time points for luciferase assay.
(0.75 MB PDF)

Figure S2 The DEYN virus remains sensitive to IFN. Huh-7.5 sh-Luc cells electroporated with either wt or DEYN J6-JFH RNA were treated with increasing amount of IFN for 3 days before cells were lysed for luciferase assay. The value of untreated samples were set to 100%.
(0.43 MB PDF)

Figure S3 Mutation of the second DY motif downstream of D316/Y317 is lethal. (A) A similar DY motif downstream of D316/Y317. (B) Replication defect of the second DY mutant. D329EY330N/J6-JFH RNA (DEYN-II) was electroporated into Huh-7.5 sh-Luc and sh-A161 cells, and luciferase assays were performed at the indicated time points.
(0.47 MB PDF)

Figure S4 Lack of correlation between the phosphorylation status of NS5A and CyPA-independence. (A) Both p56 and p58 of NS5A protein bound to CyPA. The binding reactions were performed as described in Figure 3B with His-tagged CyPA and the two forms of NS5A were resolved on a 12% SDS-PAGE. (B) DEYN mutations or CsA treatment does not change the ratio of p58 versus p56. JFH-1 or DEYN virus infected cells were treated with 4 µg/ml CsA. Cells were collected 22 hrs after the treatment and lysate was analyzed on western blot.
(0.45 MB PDF)

Figure S5Chemical shift perturbation plot for binding of wt and DEYN peptides. Perturbations in amide chemical shift were calculated as 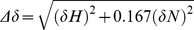, where dH (dN) represents the change in chemical shift in the H (N) dimension in parts per million. Values for wt peptide are shown as negative values for ease of viewing. The change in chemical shift indicates a change in the magnetic environment upon addition of the peptide ligand.(0.50 MB PDF)

Figure S6 DEYN mutations confer CsA resistance to a NS3-NS5B subgenomic replicon of JFH-1. Stable replicon cells containing either the WT or the DEYN mutant NS3-NS5B replicons were treated with increasing amount of CsA for 4 days before total RNA were extracted for quantitative RT-PCR to measure both HCV and GAPDH RNA levels.
(0.45 MB PDF)

Figure S7 Sh-A161 specifically inhibit expression of CyPA, but that of other CyP isoforms. Total RNA from Huh-7.5 sh-Luc and sh-A161 cells were extracted and subjected to semi-quantitative RT-PCR to analyze the expression level of human CyP isoforms A through H. Primer sequences for all the CyPs are available upon request.
(0.47 MB PDF)
