# Supplementary figures and images for: A Major Determinant of Cyclophilin Dependence and Cyclosporine Susceptibility of Hepatitis C Virus Identified by a Genetic Approach
Source: PLoS Pathog. 2010 Sep 23;6(9):e1001118. doi: 10.1371/journal.ppat.1001118 (PMC2944805; doi:10.1371/journal.ppat.1001118)

S1

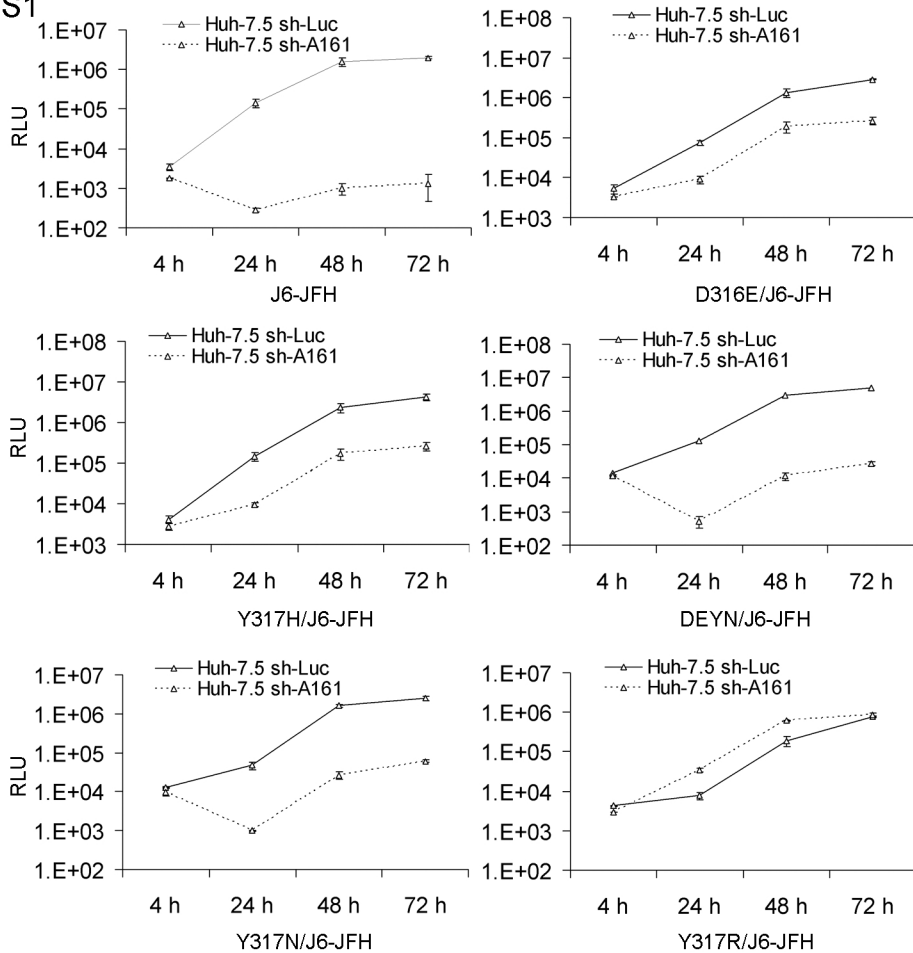

Supplement: Figure S1 — Mutations at the DY motif confer reduced CyPA dependence in the J6-JFH(p7-Rluc2A) background. Mutant RNAs were transfected into Huh-7.5 sh-Luc and sh-A161 cells by electroporation. Cells were then collected at the indicated time points for luciferase assay. (0.75 MB PDF) [file ppat.1001118.s001.pdf]

S2

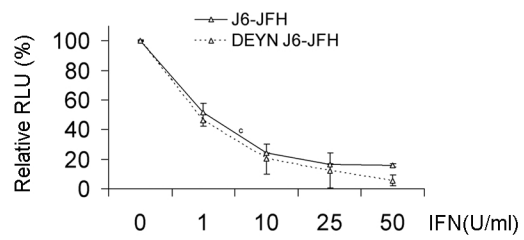

Supplement: Figure S2 — The DEYN virus remains sensitive to IFN. Huh-7.5 sh-Luc cells electroporated with either wt or DEYN J6-JFH RNA were treated with increasing amount of IFN for 3 days before cells were lysed for luciferase assay. The value of untreated samples were set to 100%. (0.43 MB PDF) [file ppat.1001118.s002.pdf]

S3

A GFPRALPAWARPDYNPPLVE  
WRRPDYQPPLVE

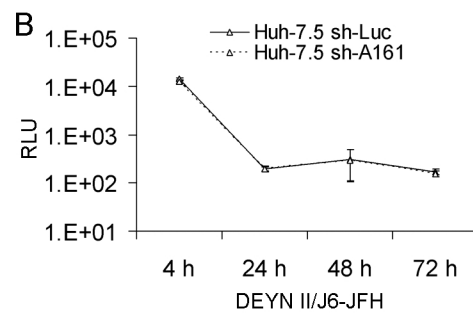

Supplement: Figure S3 — Mutation of the second DY motif downstream of D316/Y317 is lethal. (A) A similar DY motif downstream of D316/Y317. (B) Replication defect of the second DY mutant. D329EY330N/J6-JFH RNA (DEYN-II) was electroporated into Huh-7.5 sh-Luc and sh-A161 cells, and luciferase assays were performed at the indicated time points. (0.47 MB PDF) [file ppat.1001118.s003.pdf]

S4 A

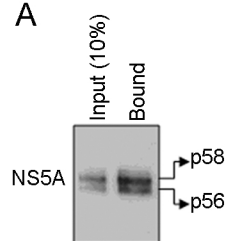

B

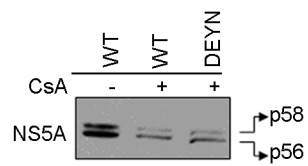

Supplement: Figure S4 — Lack of correlation between the phosphorylation status of NS5A and CyPA-independence. (A) Both p56 and p58 of NS5A protein bound to CyPA. The binding reactions were performed as described in Figure 3B with His-tagged CyPA and the two forms of NS5A were resolved on a 12% SDS-PAGE. (B) DEYN mutations or CsA treatment does not change the ratio of p58 versus p56. JFH-1 or DEYN virus infected cells were treated with 4 µg/ml CsA. Cells were collected 22 hrs after the treatment and lysate was analyzed on western blot. (0.45 MB PDF) [file ppat.1001118.s004.pdf]

S5

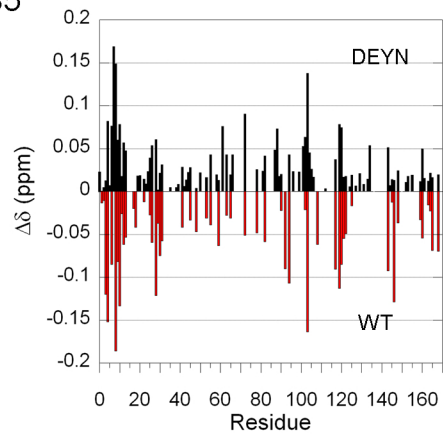

Supplement: Figure S5 — Chemical shift perturbation plot for binding of wt and DEYN peptides. Perturbations in amide chemical shift were calculated as , where dH (dN) represents the change in chemical shift in the H (N) dimension in parts per million. Values for wt peptide are shown as negative values for ease of viewing. The change in chemical shift indicates a change in the magnetic environment upon addition of the peptide ligand. (0.50 MB PDF) [file ppat.1001118.s005.pdf]

S6

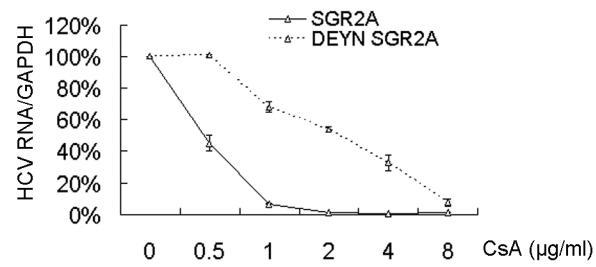

Supplement: Figure S6 — DEYN mutations confer CsA resistance to a NS3-NS5B subgenomic replicon of JFH-1. Stable replicon cells containing either the WT or the DEYN mutant NS3-NS5B replicons were treated with increasing amount of CsA for 4 days before total RNA were extracted for quantitative RT-PCR to measure both HCV and GAPDH RNA levels. (0.45 MB PDF) [file ppat.1001118.s006.pdf]

S7

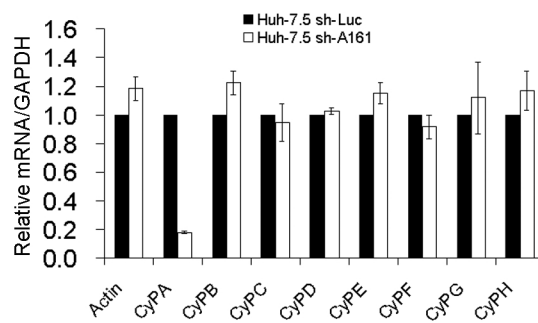

Supplement: Figure S7 — Sh-A161 specifically inhibit expression of CyPA, but that of other CyP isoforms. Total RNA from Huh-7.5 sh-Luc and sh-A161 cells were extracted and subjected to semi-quantitative RT-PCR to analyze the expression level of human CyP isoforms A through H. Primer sequences for all the CyPs are available upon request. (0.47 MB PDF) [file ppat.1001118.s007.pdf]
